# Transcriptome Profiling of the Salt Stress Response in the Leaves and Roots of Halophytic *Eutrema salsugineum*

**DOI:** 10.3389/fgene.2021.770742

**Published:** 2021-11-18

**Authors:** Chuanshun Li, Yuting Qi, Chuanzhi Zhao, Xingjun Wang, Quan Zhang

**Affiliations:** ^1^ Shandong Provincial Key Laboratory of Plant Stress Research, College of Life Science, Shandong Normal University, Jinan, China; ^2^ Bio-Tech Research Center, Shandong Academy of Agricultural Sciences, Shandong Provincial Key Laboratory of Crop Genetic Improvement, Ecology and Physiology, Jinan, China

**Keywords:** *Eutrema salsugineum*, RNA-seq, salt shock, autophagy, lignin biosynthesis, peroxisome, sugar metabolism, transcription factor

## Abstract

*Eutrema salsugineum* can grow in natural harsh environments; however, the underlying mechanisms for salt tolerance of *Eutrema* need to be further understood. Herein, the transcriptome profiling of *Eutrema* leaves and roots exposed to 300 mM NaCl is investigated, and the result emphasized the role of genes involved in lignin biosynthesis, autophagy, peroxisome, and sugar metabolism upon salt stress. Furthermore, the expression of the lignin biosynthesis and autophagy-related genes, as well as 16 random selected genes, was validated by qRT-PCR. Notably, the transcript abundance of a large number of lignin biosynthesis genes such as *CCoAOMT*, *C4H*, *CCR*, *CAD*, *POD*, and *C3′H* in leaves was markedly elevated by salt shock. And the examined lignin content in leaves and roots demonstrated salt stress led to lignin accumulation, which indicated the enhanced lignin level could be an important mechanism for *Eutrema* responding to salt stress. Additionally, the differentially expressed genes (DEGs) assigned in the autophagy pathway including *Vac8*, *Atg8*, and *Atg4*, as well as DEGs enriched in the peroxisome pathway such as *EsPEX7*, *EsCAT*, and *EsSOD2*, were markedly induced in leaves and/or roots. In sugar metabolism pathways, the transcript levels of most DEGs associated with the synthesis of sucrose, trehalose, raffinose, and xylose were significantly enhanced. Furthermore, the expression of various stress-related transcription factor genes including *WRKY*, *AP2/ERF-ERF*, *NAC*, *bZIP*, *MYB*, *C2H2*, and *HSF* was strikingly improved. Collectively, the increased expression of biosynthesis genes of lignin and soluble sugars, as well as the genes in the autophagy and peroxisome pathways, suggested that *Eutrema* encountering salt shock possibly possess a higher capacity to adjust osmotically and facilitate water transport and scavenge reactive oxidative species and oxidative proteins to cope with the salt environment. Thus, this study provides a new insight for exploring the salt tolerance mechanism of halophytic *Eutrema* and discovering new gene targets for the genetic improvement of crops.

## Introduction

Salt stress is a major abiotic stress that can affect the growth and development of plants and leads to reductions in crop productivity. When responding to salt stress conditions, plants require numerous biochemical and physiological alterations that accompany the expression changes of a number of stress-related genes (Bressan et al., 2009; Zhao et al., 2020). Two main groups of genes participate in the salt stress response including the stress-regulated genes encoding regulatory proteins such as transcription factors, and proteins functioning in stress tolerance such as detoxification enzymes, osmoprotectant biosynthesis, and late embryogenesis abundant proteins (LEA proteins) (Gupta and Huang, 2014; Zhao et al., 2020).

Transcription factors (TFs) are considered the most important regulators and involved in the regulation of a broad range of target genes associated with plant tolerance and adaptation by binding to the specific *cis*-acting elements in the promoters of the target genes (Zhu et al., 2010; Zhao et al., 2020). Some numbers of TF families including AP2/ERF, NAC, WRKY, MYB, bZIP, HSF, and C2H2 have been implicated in stress responses, and they are possibly associated with enhanced stress tolerance in plants (Kiełbowicz-Matuk, 2012; Scharf et al., 2012; Wang et al., 2016). For example, *Arabidopsis* bZIP24, ANAC042, MYB41, WRKY33, HSFA2, and C2H2-type zinc finger (ZAT7, ZAT10/STZ, ZAT12, AZF1, AZF2, AZF3) have been documented to play important roles in the response to salt and osmotic stress (Kiełbowicz–Matuk, 2012; Golldack et al., 2014). Abscisic acid (ABA) is a vital cellular signal that modulates the expression of a number of salt and water-deficit responsive genes (Gupta and Huang, 2014). The positive relationship between ABA accumulation and salinity tolerance has been at least partially attributed to the accumulation of K^+^, Ca^2+^, and compatible solutes such as proline and sugars (Keskin et al., 2010).

In general, the enhanced lignification of cell walls, thereby facilitating water transport and providing stress resistance (Hu et al., 2019; Dong and Lin, 2021), has been observed in plants exposed to salt stress (Neves et al., 2010; Chun et al., 2019). A branch of the phenylpropanoid pathway is responsible for the synthesis of lignin, and many enzymes in the lignin biosynthetic pathway exhibit extensive substrate specificity (Xie et al., 2018). Phenylalanine ammonia–lyase (PAL) is the first committed enzyme in the phenylpropanoid pathway leading to the production of lignin, lignans, and flavonoids (Saito et al., 2013). Cinnamic acid 4-hydroxylase (C4H) is a cytochrome P450 monooxygenase which catalyzes the hydroxylation of *trans*-cinnamic acid at C-4 position to yield *p*-coumaric acid (4-coumaric acid) (Saito et al., 2013). The activated reaction of *p*-coumaric acid is catalyzed by 4-coumaric acid: CoA ligase (4CL) (Saito et al., 2013). The *p*-coumaroyl shikimate 3′-hydroxylase (C3′H) and hydroxycinnamoyl CoA: shikimate/quinate hydroxycinnamoyl transferase (HCT) catalyze the synthesis of the shikimate and quinate esters of *p*-coumaric acid, which leads to the biosynthesis of guaiacyl (G) and syringyl (S) lignin units. Lignin is composed of three monomers known as hydroxyphenyl (H), guaiacyl, and syringyl that are derived from *p*-coumaryl alcohols, coniferyl alcohols, and sinapyl alcohols, respectively (Dong and Lin, 2021). The enzymes of caffeoyl CoA O-methyltransferase (CCoAOMT), caffeate 3-O-methyltransferase (COMT), ferulate 5-hydroxylase (F5H), cinnamoyl CoA reductase (CCR), and cinnamyl alcohol dehydrogenase (CAD) are involved in the biosynthesis of *p*-coumaryl alcohols, coniferyl alcohols, and sinapyl alcohols (Yang et al., 2019). Additionally, peroxidase (POD/POX) and laccase (LAC) participated in the polymerization of monolignols to yield the lignin polymer as a final product (Xie et al., 2018).

Reactive oxidative species (ROS) accumulation can be enhanced by salinity stress, which leads to oxidative damages in various cellular components, and interrupt vital cellular functions of plants (Apel and Hirt, 2004; Asada, 2006; Van Breusegem and Dat, 2006). Autophagy is involved in degrading oxidized proteins, in which cytoplasmic components are taken up into a vacuole or lysosome for degradation under oxidative stress conditions (Xiong et al., 2007a; Xiong et al., 2007b). Autophagy is critical for the survival of eukaryotes and plays a central role in stress responses because it enables cells to maintain homeostasis under stressful conditions (Xiong et al., 2007b). The complexes formed by Atg8, Atg4, Atg7, and Atg3 are mainly involved in the vehicle expansion and completion process of autophagy (Ichimura et al., 2000). The ubiquitin-like protein Atg8 is activated by Atg7, transferred to E2 enzyme Atg3, and finally conjugated to phosphatidylethanolamine (PE). Atg4 mediates both the C-terminal processing and deconjugation of Atg8 (Ichimura et al., 2000). Vac8 genes are related to autophagy initiation; they coordinate the site of autophagosome formation with vacuole fusion and improve the efficiency of autophagy by localizing autophagosome formation within the cell (Hollenstein et al., 2019). In the peroxisome pathway, the function of Mpv17, CAT, and SOD is related to ROS metabolism and could contribute to the stress tolerance of plants (Wang et al., 2004; Wi et al., 2020). Peroxisomes, as a metabolic organelle, typically contain enzymes involved in the lipid metabolism, and the catalase convert H_2_O_2_ to water and O_2_ (Van den Bosch et al., 1992; Wanders et al., 2001). HPCL2 was involved in the α-oxidation of fatty acid (Foulon et al., 1999), while ACOX1 and ACAA1 were related to the β-oxidation of fatty acid (Arai et al., 2008).

Starch is a key molecule for modulating plant responses to abiotic stresses, such as water deficit and high salinity (Thalmann and Santelia, 2017). When photosynthesis is limited, starch is generally remobilized, and released sugars and other derived metabolites provide energy for plant growth under challenging environmental conditions (Thalmann and Santelia, 2017). Accumulation of sugars (glucose, sucrose, trehalose, xylose, and raffinose) can function as osmoprotectants under salt stress, and it was observed that salt stress increases the level of reducing sugars in the cells of different plants (Krasensky and Jonak, 2012; Thalmann and Santelia, 2017). Sucrose, a main sugar accumulated through photosynthesis, may serve as an important source of carbon and energy and also as osmotic protectants to enhance tolerance to abiotic stress (Ruan, 2014). Enzymes like sucrose synthetase (SS), sucrose phosphate synthetase (SPS), and invertase (INV) are tightly involved in the sucrose metabolism (Krasensky and Jonak, 2012). Trehalose is present in trace amounts in most angiosperms, and abiotic stress can increase the accumulation of trehalose to improve the salinity tolerance of plants (Garg et al., 2002). An increase in trehalose levels was observed when trehalose-6-phosphate synthase (TPS) and trehalose-6-phosphate phosphatase (TPP) were expressed in rice, and the transgenic plants showed an increased tolerance to various abiotic stresses (Garg et al., 2002). Additionally, raffinose, suggested to act as compatible solute and antioxidant and serve as signal in response to stress, is associated with the defense mechanism of stress tolerance (ElSayed et al., 2014). In the halophyte of *Eutrema*, it is reported that more raffinose and other raffinose family oligosaccharides accumulated than in *Arabidopsis* during salt stress (Taji et al., 2004).

Late embryogenesis abundant (LEA) proteins are descripted as a multifunctional stress protein that maintains normal metabolism under severe conditions (Close, 1996). No tissue-specific *LEA* gene expression has been considered as one main regulatory mechanism developed against dehydration caused by drought and salt (Tunnacliffe and Wise, 2007). In a previous study, 148 putative LEA protein sequences of *Eutrema* were obtained by aligning them with *Arabidopsis* LEA proteins, and 52 LEA protein sequences exhibited high sequence similarity with the orthologs in *Arabidopsis* (Lee et al., 2013).

*Eutrema salsugineum* (salt cress) represents an extremophyte model system closely related to *Arabidopsis*, and it is tolerant to high salt, drought stress, heat stress, and cold stress (Inan et al., 2004; Taji et al., 2004; Gong et al., 2005). Stomata of salt cress are less open and more tightly closing, the leaves are more succulent-like, and its roots develop an extra endodermis and cortex cell layer relative to *Arabidopsis* during extreme salt stress (Inan et al., 2004). Compared with *Arabidopsis*, the detrimental effects of the salt on photosynthetic activity and stomatal conductance of *Eutrema* leaves were to a much less extent, and its rosette leaves exhibited more efficient protective mechanisms against Na^+^ metabolic toxicity (M'Rah et al., 2007). In addition, NaCl treatment enhanced the tonoplast (TP) and plasma membrane (PM) H^+^-transport and the hydrolytic activity of H^+^-ATPases and also greatly stimulated activity of tonoplast Na^+^/H^+^ exchange both in *Eutrema* leaves and roots (Vera–Estrella et al., 2005). Previously, the global gene expression profiling of *Eutrema* was performed by using a microarray platform and *Arabidopsis* homologous gene probes and was mainly involved in the comparative salt tolerance and stress-related genes between *Eutrema* and *Arabidopsis* (Taji et al., 2004; Gong et al., 2005; Wong et al., 2006). And these research studies showed that *Eutrema* plants are “primed” for stress, implying plants thriving in harsh stress conditions may achieve protection by constitutive activation of stress-related genes (Gong et al., 2005; Amtmann, 2009). In view of salt cress as a halophyte, it usually grows well in 200 mM NaCl, but it has a certain degree of growth inhibition in 300 mM NaCl (Inan et al., 2004). In the study, *Eutrema* were used to gain global transcriptional changes in response to 300 mM NaCl for 24 h. The result showed that the genes of several stress-relative pathways were significantly enriched in the halophyte when exposed to salt shock. The salt environment–induced genes were mainly involved in lignin biosynthesis and accumulation of soluble sugars, as well as genes in autophagy and peroxisome pathways, suggesting that the enhanced lignin accumulation and osmotic protection, and degrading oxidized proteins and scavenging ROS may be the important aspects of salt cress to respond to a salt environment.

## Materials and Methods

### Plant Materials and Salt Treatment

Seeds of *Eutrema* (Shandong ecotype) were surface-sterilized and planted on a 1/2 MS medium. Plates were kept at 4°C for 7 days to synchronize the germination of seeds and then transferred to a growth chamber with 16-h light at 26°C with a light intensity of 3,000 lux and 8 h dark at 22°C (Wang et al., 2017). Seven-day-old seedlings were transferred to soil in a growth chamber with a 16-/8-h (22/18°C) day/night cycle for 5 weeks. The seedlings were irrigated with 300 mM NaCl solution for the salt-treated plants. The control seedlings were irrigated with water. After 24 h of salt treatment, leaves and roots of the control and salt-treated seedlings were separately collected, frozen immediately in liquid nitrogen, and stored at −80°C. Leaves and roots from nine individual seedlings were collected, respectively, as one independent biological replicate to minimize the variation of samples. And two biological replicates were used for RNA-seq, which was based on the data of RNA-seq from two biological replicates that were relatively accurate and credible if there is a sufficient sequencing depth (Tarazona et al., 2015; Schurch et al., 2016).

To verify the *Eutrema* transcriptome expression profiles in response to salt shock, the same aforementioned *Eutrema* plants under 300 mM NaCl treatment for 24 h were used to collect samples from the separated leaves and roots, respectively, and the samples collected from plants under the normal growth condition were used as controls. All samples were frozen immediately in liquid nitrogen and kept at −80°C for total RNA extraction. The qRT-PCR assay was conducted using three biological replicates, each consisting of three technical replicates in leaf and root tissues, respectively.

### Library Construction and Illumina Sequencing

Total RNA was isolated from the leaves and roots using RNAiso Reagent (Takara, China) according to the manufacturer’s instructions, and treated with DNase I to eliminate DNA contamination. Magnetic beads with Oligo (dT) were used to isolate and enrich the mRNA from total RNA, and the mRNA mixed with fragmentation buffer was cleaved into about 200-bp fragments. Then the first-strand cDNA was generated from the mRNA templates using reverse transcriptase and random hexamer primers. Subsequently, the second-strand cDNA was synthesized using first-strand buffer, dNTP, DNA polymerase I, and RNase H. The double-stranded cDNA was purified with magnetic beads, and the short fragments were subsequently connected with poly(A) adapters. Finally, the suitable fragments were PCR-amplified for the cDNA library construction. In the study, eight cDNA libraries of *Eutrema* including the leaves and roots without (CKL-1, CKL-2, CKR-1, and CKR-2) or with 300 mM NaCl treatment (NaClL-1,NaClL-2, NaClR-1, and NaClR-2) for 24 h were sequenced using the Illumina HiSeq™ 2000 platform at Beijing Genomics Institute (BGI).

### Mapping of RNA Sequencing Reads and Gene Expression Analysis

Clean reads were obtained by removing the low-quality reads, adapter contamination, reads containing a large number of unknown bases (N) from the raw data using Cutadapt software (Martin, 2011). Then the clean reads were mapped to the reference genome of *Eutrema* using SOAP2 program based on no more than two mismatched bases in the alignment (Li et al., 2009). The transcripts were calculated and normalized to reads per kilobase per million reads (RPKM), representing the gene expression levels. The differentially expressed genes (DEGs) were identified by NOIseq software that offered a differentially expressed analysis method based on the nonparametric statistics for two replicated RNA-seq (Tarazona et al., 2011; Tarazona et al., 2015). The absolute values of log2FoldChange ≥1 and q-value ≥0.8 were used as the thresholds to judge the significance of gene expression differences (Tarazona et al., 2011).

### Gene Ontology and Kyoto Encyclopedia of Genes and Genomes Enrichment Analysis of Differentially Expressed Genes

Gene annotations and functional enrichment analysis, including the Gene Ontology (GO) and Kyoto Encyclopedia of Genes and Genomes (KEGG) biological pathways, were used to identify which DEGs from root and leaf tissues were significantly enriched in the GO terms and KEGG pathways. Blast2GO program was used to annotate the GO terms of DEGs against GO database (http://geneontology.org/), and WEGO web-based tool was employed to visualize the functional categories of BP (biological processes), MF (molecular functions), and CC (cellular components) (https://www.blast2go.com/, https://wego.genomics.cn/) (Conesa et al., 2005; Ye et al., 2006). In the KEGG enrichment analysis, DEGs were mapped to specific biochemical pathways against the KEGG database (http://www.genome.jp/kegg) (Kanehisa and Goto, 2000).

### Transcription Factor Analysis

iTAK software was used to identify and classify plant transcription factors based on the consensus rules that mainly summarized from plnTFDB (http://plntfdb.bio.uni-potsdam.de/v3.0/) and plantTFDB (http://planttfdb.gao-lab.org/) (Zheng et al., 2016; Pérez–Rodríguez et al., 2010; Jin et al., 2017). TBtools was employed to visualize the expression and classification of transcription factors (https://github.com/CJ-Chen/TBtools) (Chen et al., 2020).

### Quantitative Real-Time PCR Validation of RNA-Seq Result

To confirm the reliability of the RNA-seq result, quantitative real-time PCR (qRT-PCR) was performed using the SYBR Green I (Roche) in the ABI7500 Real Time System (Applied Biosystems). Sixteen differentially expressed genes were randomly selected from roots and leaves for experimental validation. *Ubiquitin* (Thhalv10000782m) was used as an internal reference gene. Specific primers of genes were designed by Primer Premier 5.0 software, as shown in Supplementary Table S1. The amplification program was as follows: 94°C for 10 min, followed by 40 cycles at 95°C for 15 s, 60°C for 10 s, and 72°C 25 s. The qRT-PCR expression analysis of each gene was performed using three technical replicates (with three biological replicates).The relative expression levels of genes were calculated according to the 2^−ΔΔCT^ method (Livak and Schmittgen, 2001). The cor.test function of the R base package was used to calculate Pearson’s correlation coefficient (PCC) between qRT–PCR (–ΔΔCT) and RNA-seq results (log_2_FC).

### Extraction and Determination of Lignin

To investigate the *Eutrema* lignin content affected by salt stress, the plants were treated with 300 mM NaCl once every 2 days, and the leaves and roots from 10-day salt-treated plants were, respectively, collected. The samples watered for 10 days were used as controls. The leaves or roots from six plants were crushed and pooled to obtain three technical replicates, and three biological replicates were employed in the study.

The extraction of lignin in the separated leaves and roots of *Eutrema* was assayed using the method referring to the protocol of Qiyi Biotechnology (Shanghai) Co., Ltd. The absorbance of the samples was determined at 280 nm with a spectrophotometer (UV-1800, Shimadzu), and glacial acetic acid was used as a blank control. The lignin content (mg/g DW) was calculated based on the formula 0.9996 × (A280 of sample − A280 of blank sample)/(A280 of standard sample − A280 of blank sample)/dry weight (g), where the sample indicates the extraction of lignin from the leaves or roots, the blank sample indicates the reaction solution without sample, and the standard sample represents the lignin standard sample presented by Qiyi.

### Statistical Analysis

Statistical significance was performed using Student’s *t*-test by R base package. The mean values and standard deviations (SDs) were calculated from three biological and three technical replicates, and significant differences relative to the controls are indicated at *p ≤ 0.05, **p ≤ 0.01, and ***p ≤ 0.001.

## Results

### RNA Sequencing Analysis in Leaf and Root Tissues Upon Salt Shock

**FIGURE 1:**
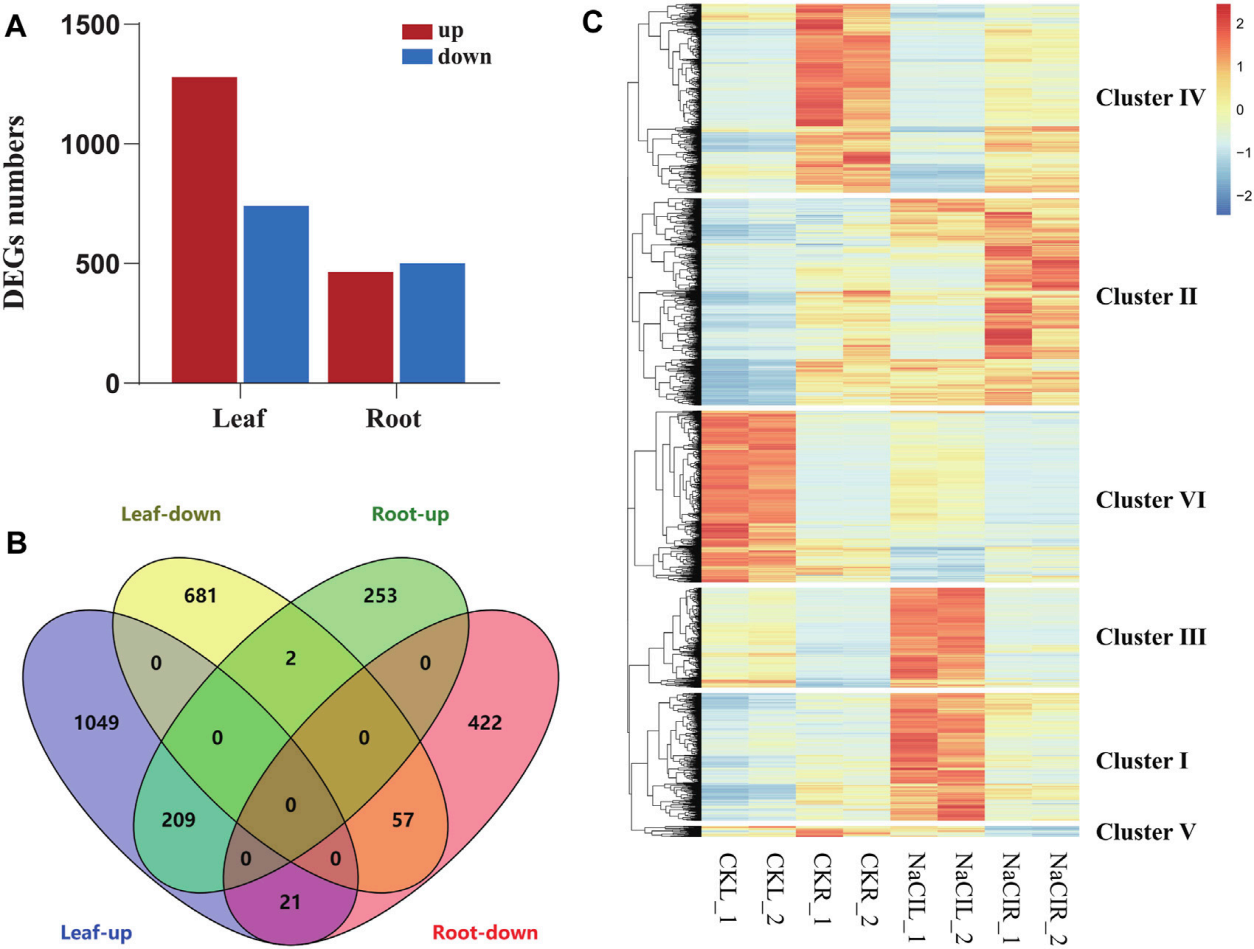
Analysis of differentially expressed genes in *Eutrema* upon 300 mM NaCl. **(A)** Numbers of differentially expressed genes (DEGs). **(B)** Venn diagram demonstrated the common and specific DEGs in roots and leaves. **(C)** Heatmap demonstrated the distinguished expression patterns of DEGs. The transcript levels calculated as RPKM are shown in the **color** legend.

*Eutrema salsugineum* is an extremophile native to harsh environments and can reproduce after exposure to 500 mM NaCl (Inan et al., 2004). Salt cress could retain normal growth in 200 mM NaCl for a long time (Inan et al., 2004). The plant leaves and roots follow distinct developmental trajectories to adapt to fluctuating environments that accompany the expression change of a number of stress-related genes (Min et al., 2020). To obtain an overview of the salt response genes in *Eutrema*, the transcriptome of leaves and roots with or without 300 mM NaCl treatment for 24 h was investigated by the Illumina Hiseq2000 platform. After removing the adapter, poly-N, and low-quality reads, clean data represented more than 99% of the raw data (Table 1). Regarding the clean data, the total number of reads was 96, 558, 016, with an average of ∼12 million reads per libraries (Table 1). Approximately 85.3% reads from leaves and 82.7% reads from roots could be mapped to the reference genome of *Eutrema*, of which about 79.9 and 78.4% reads from the leaves and roots were uniquely mapped to the reference genome, respectively (Table 1). A total of 2,019 and 964 differentially expressed genes (DEGs) were respectively identified from the leaves and roots (Figure 1A, Supplementary Table S2). More salt-responsive genes were found in leaves than that in roots, and the majority of DEGs showed different response patterns between the leaves and roots. Under salt stress condition, there were 1,279 upregulated genes in leaves, of which only 209 genes were also upregulated in roots, while 964 downregulated genes in roots, of which only 57 genes were also downregulated in leaves (Figure 1B). Our results demonstrated that 266 DEGs showed the same response trend upon salt stress, including 209 upregulated and 57 downregulated DEGs in both leaves and roots. Additionally, 23 DEGs showed the opposite expression trends between leaves and roots. For example, selenium-binding protein SBP2 (Thhalv10025040m) and plant defensin protein PDF1.2 (Thhalv10000535m) were upregulated in the roots but downregulated in the leaves, while 21 DEGs such as AP2/DREB26 (Thhalv10008639m) and cytochrome P450 (Thhalv10013359m) were downregulated in the roots but upregulated in the leaves (Figure 1B).

**TABLE 1:** Summary of reads mapping to reference genome.

Sample	Total clean reads	Total mapped reads	Unique match	Multi-position match	Unmapped reads
CKL-1	11,839,336	10,114,688 (85.43%)	9,414,799 (79.52%)	699,889 (5.91%)	1,724,648 (14.57%)
CKL-2	12,464,847	10,632,404 (85.30%)	9,964,811 (79.94%)	667,593 (5.36%)	1,832,443 (14.70%)
NaCIL-1	12,537,207	10,687,486 (85.25%)	10,038,754 (80.07%)	648,732 (5.17%)	1,849,721 (14.75%)
NaCIL-2	11,858,487	10,124,550 (85.38%)	9,508,935 (80.19%)	615,615 (5.19%)	1,733,937 (14.62%)
CKR-1	12,135,076	10,040,551 (82.74%)	9,580,615 (78.95%)	459,936 (3.79%)	2,094,525 (17.26%)
CKR-2	11,814,795	9,768,993 (82.68%)	9,311,121 (78.81%)	457,872 (3.88%)	2,045,802 (17.32%)
NaCIR-1	12,152,616	9,946,700 (81.85%)	9,336,465 (76.83%)	610,235 (5.02%)	2,205,916 (18.15%)
NaCIR-2	11,755,652	9,814,489 (83.49%)	9,281,846 (78.96%)	532,643 (4.53%)	1,941,163 (16.51%)

To analyze the differential expression patterns of all 2,696 DEGs in detail, we conducted the hierarchical clustering analysis of these genes by a heatmap, and six major clusters were distinguished (Figure 1C, Supplementary Table S2). Cluster I and cluster III contained 427 and 333 genes, respectively, which were highly induced by NaCl in leaves, such as lignin biosynthesis-related genes [*CCR* (Thhalv10014117m, Thhalv10025830m) and *COMT* (Thhalv10007957m) in cluster I; *HCL* (Thhalv10000635m), *C3′H* (Thhalv10017418m), and *CCR* (Thhalv10017002m) in cluster III] (Figure 1C, Supplementary Table S2). Cluster II consisted of 692 genes, and most of them showed an increased expression in roots and leaves when exposed to salt stress condition, including some genes encoding LEA proteins (Thhalv10019035m and Thhalv10009011m). Cluster IV contained 632 genes, and most transcripts in the cluster were significantly reduced in roots upon salt stress. In cluster V, all 36 genes were strikingly downregulated in roots, of which three genes (Thhalv10000931m, Thhalv10004985m, and Thhalv10005285m) belong to the members of AP2/ERF-ERF TF families. Furthermore, 575 genes in cluster VI, showed a similar expression pattern with reduced expression in leaves when responding to salt, of which the genes (Thhalv10010685m, Thhalv10025982m, and Thhalv10025711m) were involved in photosynthesis (Figure 1C, Supplementary Table S2).

### Gene Ontology and Kyoto Encyclopedia of Genes and Genomes Enrichment Analysis of DEGs

**FIGURE 2:**
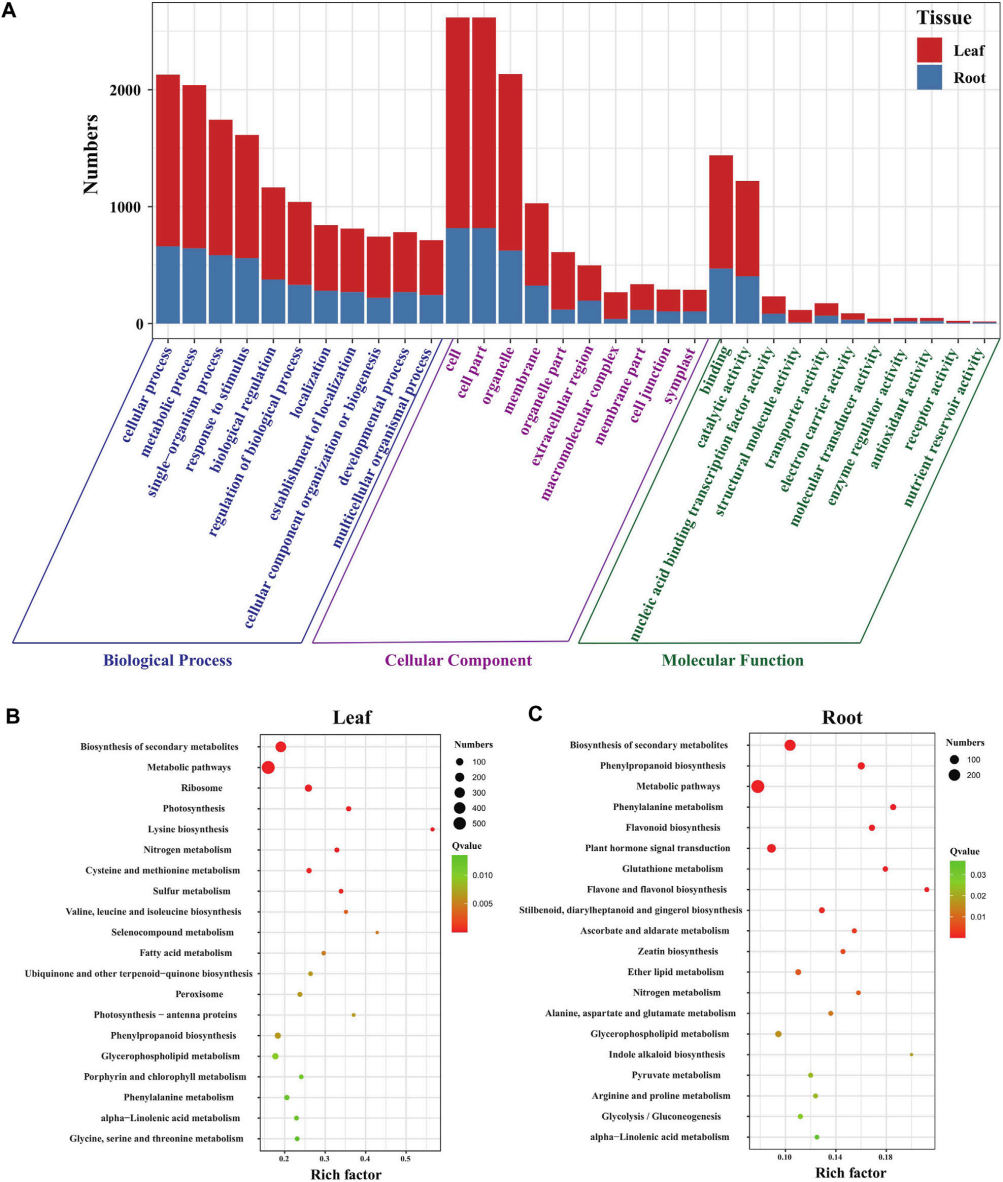
Function analysis of DEGs in *Eutrema* under salt stress. **(A)** Histogram of GO terms in leaves and roots of *Eutrema*. **(B)** Top 20 KEGG pathways in leaves. **(C)** Top 20 KEGG pathways in roots. The rich factor is the ratio of numbers of DEGs to a total of annotated genes in a certain pathway. The color of dots represents the significance of DEGs in specific pathways, and the smaller q-value indicates higher significance. The size of dots indicates the numbers of DEGs enriched.

To understand the functions of DEGs in the leaves and roots of *Eutrema* seedlings under salt stress, GO terms of DEGs were annotated and analyzed. The result showed that DEGs were enriched in “biological processes (BP),” “cellular components (CC),” and “molecular functions (MF),” including 51 GO terms in leaves and 49 GO terms in roots. Furthermore, the number of GO terms in BP, CC, and MF were 22, 17, and 12 in leaves, 22, 14, and 13 in roots, respectively (Figure 2A, Supplementary Table S3). Moreover, the GO terms, including “cellular process,” “metabolic process,” “single-organism process” and “response to stimulus” in BP, “cell,” “cell part,” “organelle,” and “membrane” in the CC and “binding,” “catalytic activity,” and “nucleic acid binding transcription factor activity” in the MF category, were the top terms that significantly enriched in both leaves and roots, which suggest a high degree of changes occurring in metabolic activity, in response to stimulus, membrane and the binding activity upon salt stress in *Eutrema* (Figure 2A).

To investigate the functional classification of DEGs in *Eutrema* upon salt stress, the enrichment analysis of KEGG pathways was performed. The result demonstrated that a total of 1710 DEGs were assigned to 126 KEGG pathways including 122 KEGG pathways in leaves, and 114 pathways in roots (Supplementary Table S4). Of which, 32 and 21 pathways were significantly enriched, respectively, in leaves and roots (*p*-value< 0.05), and the most represented top 20 pathways are shown in Figures 2B,C. In the top 20 pathways, five pathways of “biosynthesis of secondary metabolites,” “metabolic pathways,” “phenylpropanoid biosynthesis,” “glycerophospholipid metabolism,” and “phenylalanine metabolism” were shared in leaves and roots; the specific pathways of “ribosome,” “photosynthesis,” “photosynthesis-antenna proteins,” and “porphyrin and chlorophyll metabolism” were the most represented pathways in leaves (Figure 2B), whereas “flavonoid biosynthesis,” “flavone and flavonol biosynthesis,” “glutathione metabolism,” and “ascorbate and aldarate metabolism” pathways were the most represented pathways in roots (Figure 2C). The result indicated that salt stress markedly affected a variety of metabolic processes both in leaves and roots, and photosynthesis in leaves and non-enzymatic antioxidant pathways in roots.

### Validation of RNA-Seq Result Using qRT-PCR

**FIGURE 3:**
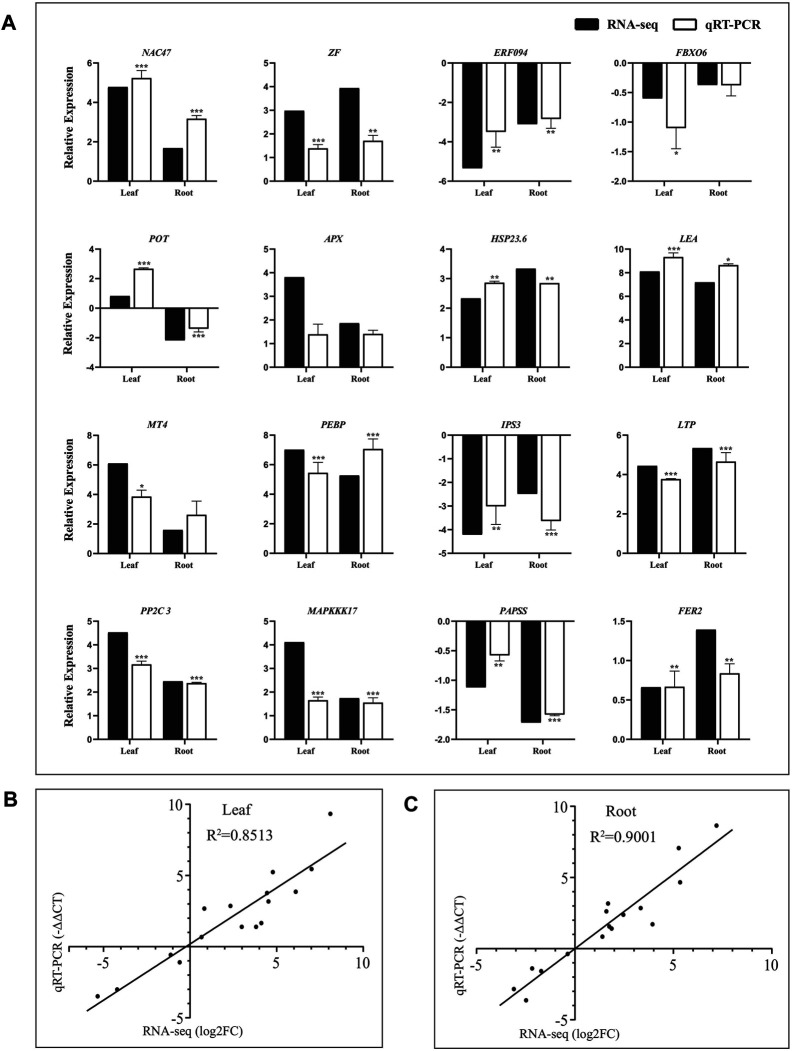
Validation of RNA-seq result using quantitative real-time PCR. **(A)** Expression verification of 16 genes in RNA-seq by qRT-PCR. Black bars indicate log_2_FC of DEGs in RNA-seq, and white bars show −ΔΔCT of DEGs in qRT–PCR. Correlation analysis of results between RNA-seq and qRT–PCR in leaves **(B)** and roots **(C)**. The data of log_2_FC (*Y*-axis) were obtained by RNA-seq, and -ΔΔCT (*X*-axis) was analyzed by qRT-PCR. The *Ubiquitin* gene (Thhalv10000782m) was used as an internal control. Each set of data were obtained from three repeats.

To confirm the reliability of the RNA-seq data, sixteen DEGs were randomly selected from the leaves and roots of *Eutrema* for qRT-PCR validation. As shown in Figure 3A, these genes represent diverse functional categories and various transcript levels, including the coding genes of transcription factors (NAC47, ERF094, zinc finger protein, and F-box protein FBX), stress-related proteins (potassium transporter POT, APX, LEA, HSP23.6, and Ferritin2), lipid metabolism–related proteins (phosphatidylethanolamine-binding protein PEBP and inositol-3-phosphate synthase IPS3, LTP), phosphorylation-related proteins (PP2C3, MAPKKK17 and 3′-phosphoadenosine 5′-phosphosulfate synthase PAPSS), and metallothionein type MT4 (Supplementary Table S5). Of which, there were 11 upregulated and 4 downregulated genes both in leaves and roots, and only the *EsPOT* gene existed oppositely expressed trend between the leaves and roots upon salt stress (Supplementary Table S5).

The relative expression levels (log_2_NaCl/CK) of 16 genes in RNA-seq were validated using RT–PCR (-ΔΔCT), and the results showed that there was good consistency on the expression levels of these genes analyzed both in leaves (*R* ^2^ = 0.8513) and roots (*R* ^2^ = 0.9001) between using qRT-PCR and RNA-seq, indicating the results of RNA-seq were trustworthy (Figure 3, Supplementary Table S5).

### Identification of Transcription factors Genes Responding to Salt Shock

**FIGURE 4:**
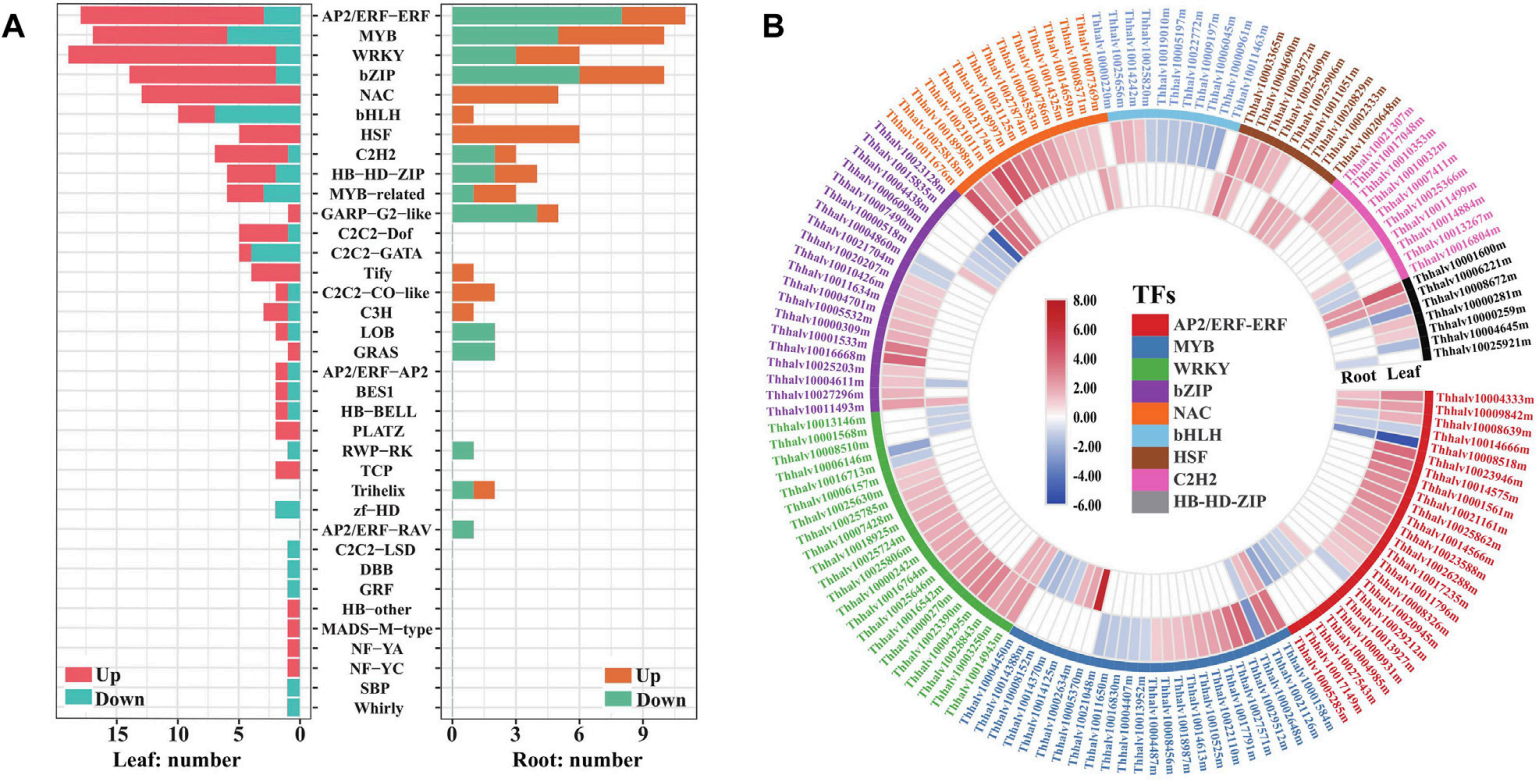
Analysis of transcription factors responding to salt stress in leaves and roots in *Eutrema*. **(A)** Number distribution of up/downregulated TF families in responsive to salt stress in leaves (left bars) and roots (right bars). The red or orange color indicates the upregulated genes, and the blue or green color indicates the downregulated genes. **(B)** Heatmap analysis of DEGs in top 9 TF families with higher numbers. The differential transcript levels calculated as log_2_ (NaCl/CK) are shown in the color legend, and the red color indicates upregulated genes, and the blue indicates downregulated genes. The various color bars represent different TF families.

Transcription factors (TFs) are central regulators of gene expression, and some members in WRKY, AP2/ERF-ERF, NAC, bZIP, MYB, HSF, and C2H2 families reported to be associated with enhanced stress tolerance in plants (Kiełbowicz–Matuk, 2012; Scharf et al., 2012; Wang et al., 2016). To provide an insight into the TF responding salt stress in *Eutrema*, the expression of TF genes annotated was analyzed and visualized with barplotting and heatmap. We found that 210 genes were annotated to 36 TF families with a significantly changed expression, including AP2/ERF–ERF, MYB, WRKY, bZIP, NAC, bHLH, HSF, and C2H2 during salt stress (Figure 4A, Supplementary Table S6). Among which, the expression of 134 and 36 TF genes were increased more than two-fold during salt stress, respectively, in leaves and roots. These upregulated TF genes mainly include WRKY (17), AP2/ERF–ERF (15), NAC (13), bZIP (12), MYB (11), C2H2 6) and HSF 5) genes in leaves, and HSF (6), NAC 5), and MYB 5) genes in roots (Figure 4A, Supplementary Table S6).

It is worth emphasizing that all NAC and HSF transcript levels in DEGs were found to increase, both in leaves and roots after salt treatment. For example, *EsRD26* (Thhalv10025818m) and *EsAF1* (Thhalv10008371m) were upregulated both in roots and leaves, while the transcript level of *EsNAC47* (Thhalv10021011m), *EsNAC3* (Thhalv10021174m), *EsNAP* (Thhalv10018997m), and *EsNAC46* (Thhalv10021125m) increased more than seven-fold in leaves (Figure 4B, Supplementary Table S6). In HSF genes, the salt-induced expression of *EsHSF6A* (Thhalv10003365m) were up to 7.1 and 9.2 times in leaves and roots, respectively, and the expression of *EsHSF4A* (Thhalv10025409m) in leaves and *EsHSF6B* (Thhalv10020829m) in roots were also markedly induced (Figure 4B, Supplementary Table S6).

In addition, most types of TF families existed in differentially expressed patterns between the leaves and roots. Of which, numbers of upregulated AP2/ERF–ERF and bZIP were more than the downregulated genes in leaves, but there existed an opposite trend in roots (Figure 4A, Supplementary Table S6). Meantime, the expression of cytokinin response factors *CRF1* and *CRF5* genes, being AP2/ERF–ERF family members, were increased up to 13 and 6.6 times in leaves, respectively; but no differential expressions were detected in roots (Figure 4B, Supplementary Table S6). The DEGs of C2H2-Dof (5) and C2H2–GATA (5) were only found in leaves, while Trihelix (2) and AP2/ERF–RAV (1) were found only in roots (Figure 4A, Supplementary Table S6). In brief, salt stress resulted in extensive response of TF genes in AP2/ERF–ERF, MYB, bZIP, WRKY, NAC, and HSF families, and the expression patterns of most of them were different between the leaves and roots of *Eutrema*.

### Enhancement in Expression of Lignin Biosynthesis Genes and Lignin Content During Salt Stress

**FIGURE 5:**
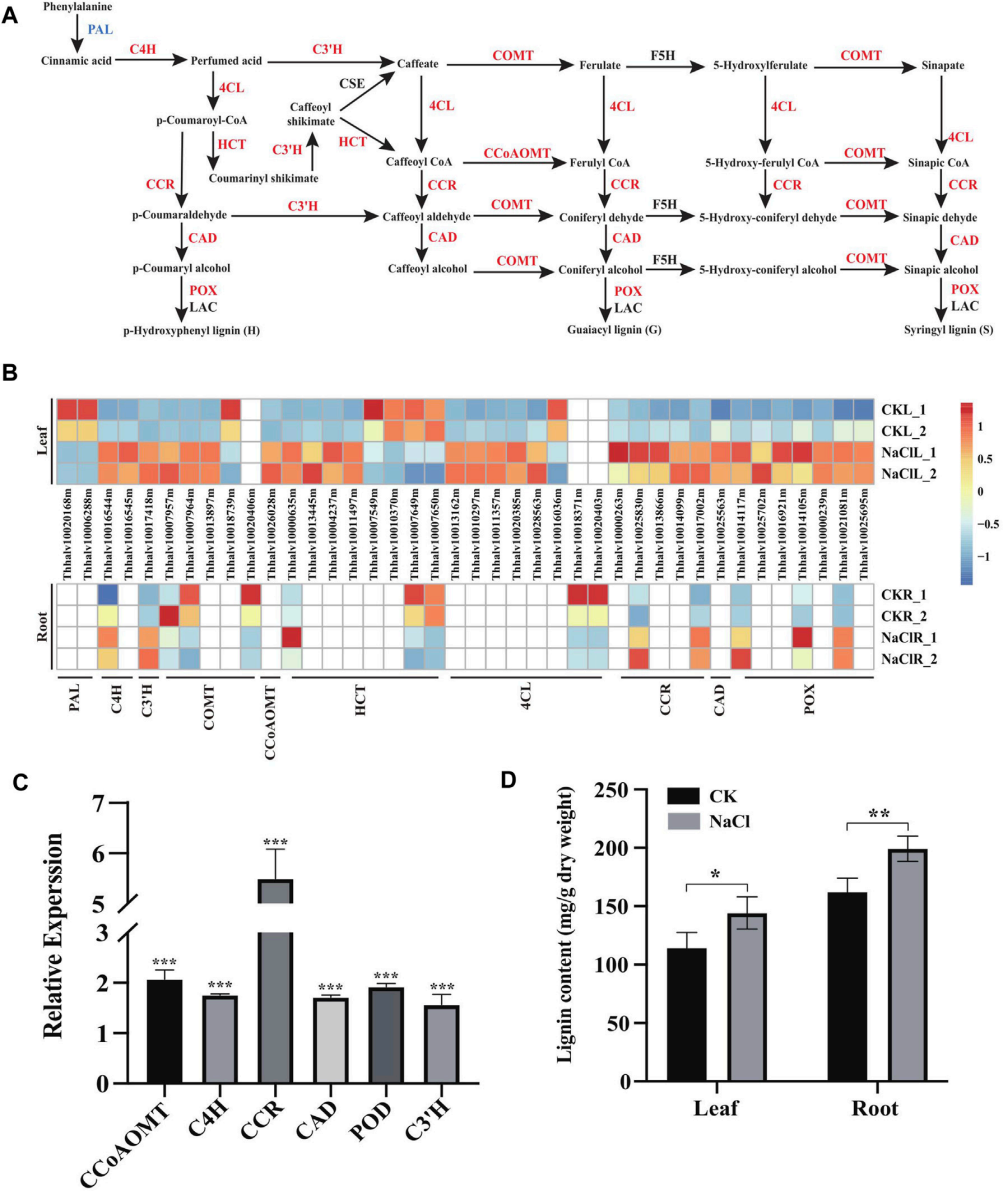
Expression analysis of DEGs enriched in the lignin biosynthesis pathway, and lignin content change under salt stress. **(A)** DEGs enriched in the lignin biosynthesis pathway in leaves. PAL, phenylalanine ammonia-lyase; C4H, cinnamic acid 4-hydroxylase; C3′H, *p*-coumaroyl shikimate 3′-hydroxylase; COMT, caffeate 3-O-methyltransferase; F5H, ferulate 5-hydroxylase; 4CL, 4-coumaric acid: CoA ligase; HCT, hydroxycinnamoyl CoA: shikimate/quinate hydroxycinnamoyl transferase; CCR, cinnamoyl CoA reductase; CAD, cinnamyl alcohol dehydrogenase; POX, peroxidase; and LAC, laccase. **(B)** Heatmap analysis of DEGs enriched in the lignin biosynthesis pathway in leaves and roots. The transcript levels calculated as RPKM are shown in the color legend. **(C)** Relative expression (2^−ΔΔCT^) of lignin biosynthesis genes in NaCl-treated *Eutrema* leaves was determined using quantitative real-time PCR. The *Ubiquitin* gene (Thhalv10000782m) was used as an internal control. Each set of data were obtained from three repeats. **(D)** Lignin content of leaves and roots in salt-treated and normal growth *Eutrema* plants.

Enhanced cell wall lignification has been observed in plants exposed to various environmental stresses (Dong and Lin, 2021). In general, NaCl may enhance lignin production that solidifies the cell wall, and the enhanced lignin biosynthesis is a critical factor in plant adaptation and tolerance to salt stress (Neves et al., 2010; Chun et al., 2019). Phenylpropanoids, derived from phenylalanine, are the pivotal metabolic precursors to synthesize the lignin monolignol (Dong and Lin, 2021). Our transcriptome demonstrated 40 DEGs including *PAL* (2), *C4H* (2), *C3′H* (1), *COMT* (5), *CCoAOMT* (1), *HCT* (8), *4CL* (8), *CCR* (5), *CAD* (1), and *POX/POD* (7) were enriched in the phenylalanine metabolism (ko00360) and phenylpropanoid (ko01904) pathways involved in lignin biosynthesis. Moreover, these gene expressions significantly changed in the leaves and/or roots of *Eutrema* upon salt stress (Figure 5A and Figure 5B, Supplementary Table S7).

In the phenylpropanoid pathway, expression of *EsC4H* (Thhalv10016545m), *EsHCT* (Thhalv10000635m), *EsC3′H* (Thhalv10017418m), and *EsCCR* (Thhalv10017002m) genes was induced both in leaves and roots, while the transcript level of *EsC4H* (1), *Es4CL* (5), *EsHCT* (3), *EsCCR* (4), and *EsCAD* (1) was markedly enhanced only in leaves that are responsive to salt (Figures 5A,B, Supplementary Table S7). Furthermore, the upregulation of *EsCCoAOMT1* (Thhalv10026028) and *EsCOMT1* (Thhalv10007957m, Thhalv10007964m, and Thhalv10013897m) genes was observed in the salt-treated leaves of plants. The transcripts of *EsCOMT* genes (Thhalv10007957m and Thhalv10007964m) were strongly enhanced in the leaf tissue by 8 and 7.5 times but reduced expression in roots, indicating that there existed the different salt stress response patterns of *EsCOMT* genes between the leaves and roots (Figures 5A,B, Supplementary Table S7). Peroxidase (POD/POX) and laccase (LAC) are involved in the polymerization of monolignols (Xie et al., 2018). In this study, salt stress resulted in induced expression of three *POX* genes (Thhalv10021081m, Thhalv10014117m, and Thhalv10014105m) both in leaves and roots. However, the expression of *EsPAL* (Thhalv10020168m and Thhalv10006288m) was declined in leaves and not markedly altered in root tissues upon salt stress (Figures 5A,B, Supplementary Table S7). Additionally, the lignin biosynthesis genes are largely regulated at the transcription level by lignin-specific transcription factor (Zhou et al., 2009). For instance, *Arabidopsis* AtMYB15 could regulate defense-induced lignification via modulating the expression of lignin synthesis genes (Chezem et al., 2017). In *Eutrema*, there was an increase in the expression level of the *EsMYB15* (Thhalv10022110m) gene up to 4.3 times in leaves upon salt stress (Supplementary Table S6), thus suggesting that *EsMYB15* was possibly an important regulatory gene concerning lignification by modulating expression of lignin biosynthesis–related genes.

To validate the expression of lignin biosynthesis genes in RNA-seq, the leaves of 300 mM NaCl-treated and normal growth plants were used to analyze the expression of *CCoAOMT*, *C4H*, *CCR*, *CAD*, *POD*, and *C3′H* genes in the lignin biosynthetic pathway by quantitative real-time PCR (Figure 5C, Supplementary Table S5). The result showed that significantly upregulated *CCoAOMT*, *C4H*, *CCR*, *CAD*, *POD*, and *C3′H* genes were observed in *Eutrema* salt-treated leaves relative to the control in the normal growth condition (Figure 5C, Supplementary Table S5). To further understand NaCl-stimulated lignin production in *Eutrema* leaves and roots, 10-day salt-treated (300 mM NaCl) plants were employed to examine the lignin content. We found that salinity stress markedly increased the lignin accumulation of *Eutrema* leaf and root tissues (Figure 5D, Supplementary Table S8). Therefore, the enhanced lignification caused by the NaCl environment, which was suggested to be an adaptation mechanism for *Eutrema* in resisting salinity-imposed stress.

Taken together, salt shock induced the expression of 40 lignin biosynthesis genes and a lignin biosynthesis–regulating gene *MYB15* in *Eutrema* RNA-seq. Combined with the verification result of qRT-PCR and the enhanced lignin content under salt stress, as well as the function of lignification in enhancing the cell wall integrity and facilitating water transport, we inferred that the lignin biosynthesis could be an important response for *Eutrema* encountering salt environment.

### Increased Expression of Genes in Autophagy and Peroxisome Pathways Upon Salt Stress

**FIGURE 6:**
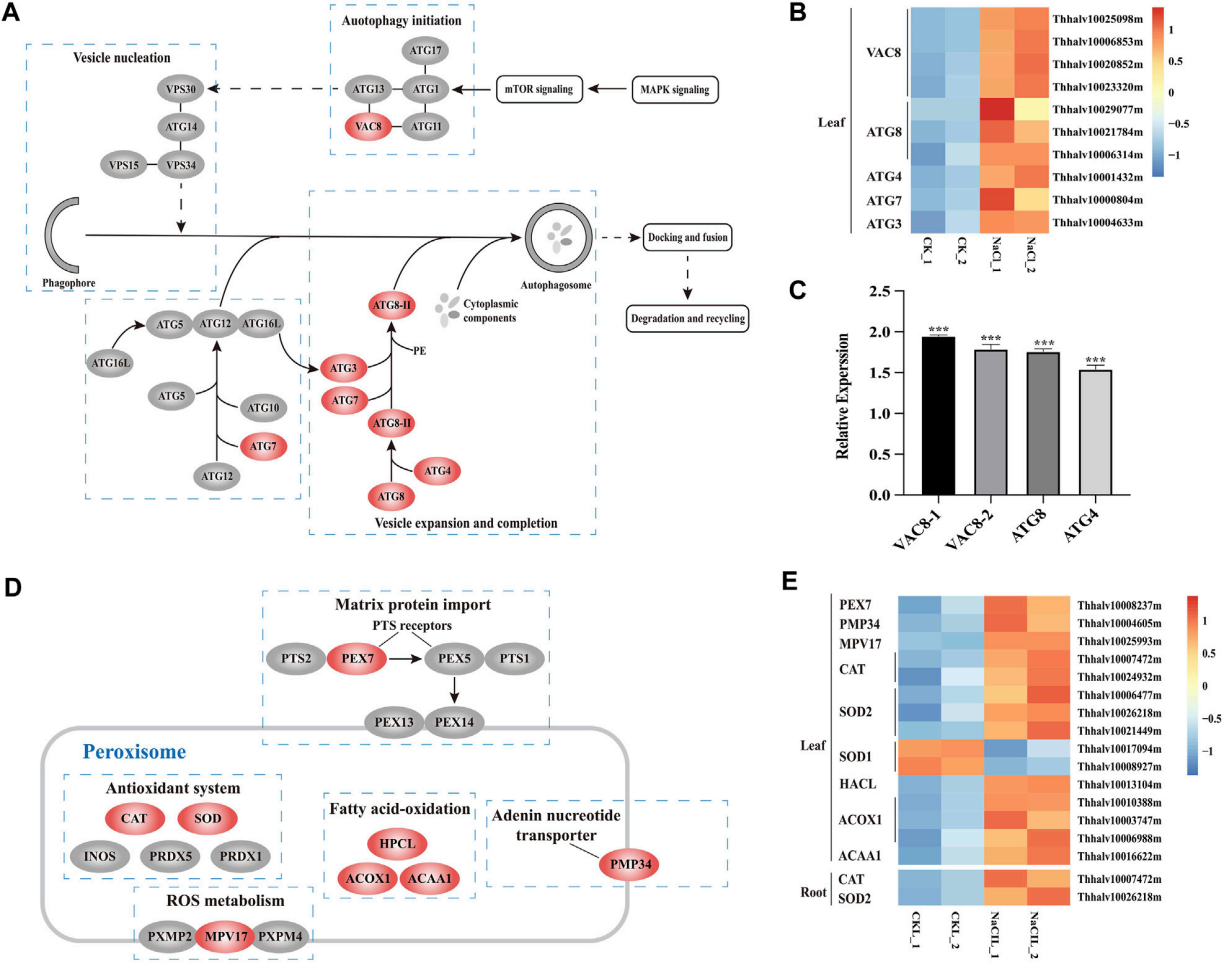
DEGs enriched in pathways of autophagy and peroxisome, and heatmap analysis of DEGs. **(A)** DEGs enriched in the autophagy pathway. MAPK, mitogen-activated protein kinase; mTOR, mammalian target of rapamycin; ATG, autophagy-related protein; VAC, vacuolar membrane protein; and VPS, vacuolar protein sorting. **(B)** Heatmap analysis of DEGs of leaves enriched in the autophagy pathway. The transcript levels calculated as RPKM are shown in the color legend. **(C)** Relative expression (2^−ΔΔCT^) of autophagy-related biosynthesis genes in NaCl-treated *Eutrema* leaves determined by qRT-PCR. The *Ubiquitin* gene (Thhalv10000782m) was used as an internal control. Each set of data were obtained from three repeats. **(D)** DEGs enriched in the peroxisome pathway. PEX, peroxin; PTS, peroxisome targeting signal; CAT, catalase; SOD, superoxidase dismutase; INOS, nitric oxide synthases; PRDX, peroxiredoxin; HPCL, 2-hydroxyacyl-CoA lyase; ACOX, acyl-coA oxidase; ACAA, acetyl-CoA acyltransferase; PMP, peroxisomal membrane protein. **(E)** Heatmap analysis of DEGs enriched in the peroxisome pathway. The transcript levels calculated as RPKM are shown in the color legend.

Salt stress can cause oxidative damage to cells, resulting in the accumulation of ROS and oxidized proteins. Autophagy plays a vital role on scavenging oxidized proteins (Xiong et al., 2007b). Atg8, Atg4, Atg7, and Atg3 proteins are involved in Atg8-mediated ubiquitination reactions, and Vac8 are associated with autophagy initiation and localize autophagosome formation within the cell (Hollenstein et al., 2019). The transcriptome profiling of *Eutrema* upon salt stress showed that the expression of ten DEGs including *EsVac8* (4), *EsAtg8* (3), *EsAtg4* (1), *EsAtg7* 1), and *EsAtg3* (1) was significantly increased in leaves (Figures 6A,B, Supplementary Table S9). To further verify the expression of autophagy-related genes in RNA-seq, we used the real-time quantitative PCR to analyze the transcript abundance of *EsVac8-1* (Thhalv10023320m), *EsVac8-2* (Thhalv10020852m), *EsAtg8* (Thhalv10021784m), and *EsAtg4* (Thhalv10001432m) genes in leaves that involved in the autophagy pathway and found that the expression of these genes checked was induced when *Eutrema* was exposed to saline conditions compared with the control (Figure 6C, Supplementary Table S5). The aforementioned genes were all enriched in the autophagy pathway (ko04140), which are mainly involved in processes of autophagy initiation, vesicle expansion, and completion. Hence, we infer that the enhanced expression of DEGs enriched in the autophagy pathway (ko04140) could contribute to the defense response of plants when *Eutrema* was exposed to saline shock.

The peroxisome pathway was involved in eliminating exceeded ROS generated by various stresses and balancing the ROS level. The proteins of PEX7 and MPV17 exist on the peroxisome membrane, of which AtMPV17 contributes to osmotic stress tolerance in plants (Wi et al., 2020), while PEX7 acts as an import receptor of peroxisomal-targeting signal PTS2 (Woodward and Bartel, 2005). In the present study, 15 DEGs existed significant changes and were enriched in the peroxisome pathway (ko04146) in leaves and/or roots in *Eutrema*, among which the transcript level of five genes were obviously upregulated in salt-treated leaves, including *EsPEX7* (Thhalv10008237m) and *EsMPV17* (Thhalv10025993m) (Figures 6D,E, Supplementary Table S10). In addition, the genes encoding antioxidant enzymes, such as *EsCAT* (Thhalv10007472m) and *EsSOD2* (Thhalv10026218m), were markedly induced both in leaves and roots. Particularly, the expression of *EsCAT* (Thhalv10007472m) was strongly enhanced by 7.5 and 4 times, respectively, in leaves and roots. However, the transcripts of *EsCAT* (Thhalv10024932m) and *EsSOD2* genes (Thhalv10006477m and Thhalv10021449m) were significantly induced only in leaves (Figures 6D,E, Supplementary Table S10). In addition, the expression level of five genes including *EsHPCL2* (Thhalv10013104m), *EsACOX1* (Thhalv10010388m, Thhalv10003747m, and Thhalv10006988m), and *EsACAA1* (Thhalv10016622m) associated with α- and β-oxidation of fatty acid, was markedly elevated in leaves (Figures 6D,E, Supplementary Table S10). Therefore, it was suggested that proteins targeted to peroxisome involved in the ROS metabolism, α- and β-oxidation of fatty acid, could be an important aspect for *Eutrema* in response to salt shock.

### NaCl Effects on the Expression of Genes Involved in Carbohydrate Metabolism

**FIGURE 7:**
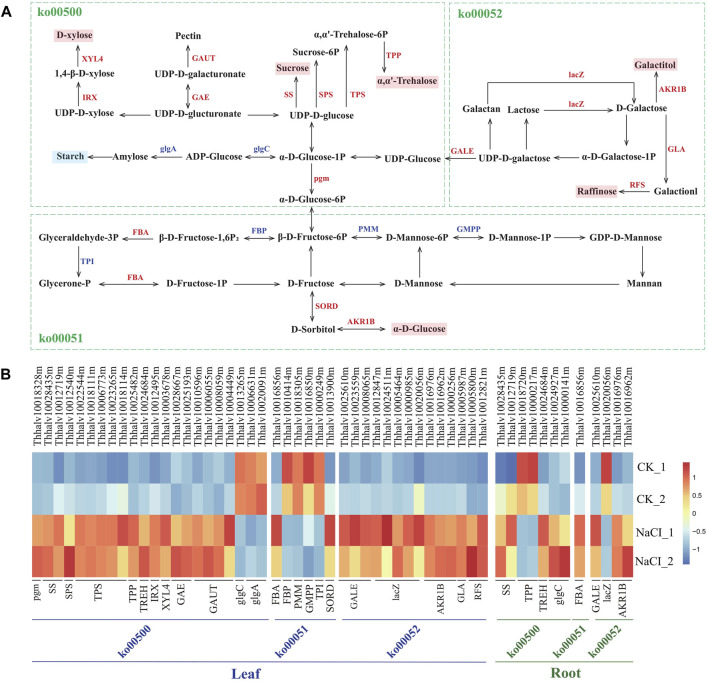
Schematic view of genes participating in starch and carbohydrate metabolism and heatmap analysis of DEGs. **(A)** The schematic view of genes participating in carbohydrate metabolism. XYL4, xylan 1,4-beta-xylosidase; IRX, 1,4-beta-D-xylan synthase; GAUT, galacturonosyltransferase; GAE, UDP-glucuronate 4-epimerase; SS, sucrose synthetase; SPS, sucrose phosphate synthetase; TPS, trehalose-6-phosphate synthase; TPP, trehalose-6-phosphate phosphatase; lacZ, beta-galactosidase; AKR1B, aldehyde reductase; GLA, alpha-galactosidase; RFS, raffinose synthase; glgA, glycogen synthase; glgC, glucose-1-phosphate adenylyltransferase; GALE, UDP-glucose 4-epimerase; pgm, phosphoglucomutase; FBA, fructose-bisphosphate aldolase; FBP, fructose-1,6-bisphosphate phosphatase; PMM, phosphomannomutase; TPI, triosephosphate isomerase; GMPP, mannose-1-phosphate guanylyltransferase; SORD, L-iditol 2-dehydrogenase. **(B)** Heatmap analysis of DEGs enriched in pathways of carbohydrate metabolism. The transcript levels calculated as RPKM are shown in the color legend.

Analysis of the genes affecting starch and carbohydrate metabolism revealed that salt stress results in transcript abundance differences of ∼45 genes in salinized *Eutrema* plants relative to controls. These DEGs were enriched in starch and sucrose metabolism (ko00500), fructose, and mannose metabolism (ko00051) and galactose metabolism (ko00052), involved in the biosynthesis of sucrose, trehalose, raffinose, xylose, pectin, degradation of starch, and the conversion among glucose, fructose, and galactitol, among which 35 DEGs markedly upregulated in leaves (Figure 7, Supplementary Table S11).

Sucrose and trehalose, serving as an osmotic protectant to stabilize proteins and membranes, protect organisms against several physical and chemical stresses, including salinity stress (Gupta and Huang, 2014; Ruan, 2014). The synthesis-related genes of sucrose and trehalose, remarkably affected by salinity, were analyzed in the research. The result showed that the expression of *SS* genes encoding sucrose synthetase (Thhalv10028435m and Thhalv10012719m) both in leaves and roots was enhanced more than three-fold by salt stress. The expression of five *TPS* genes (Thhalv10022544m, Thhalv10018111m, Thhalv10006773m, Thhalv10023265m, and Thhalv10018114m) encoding trehalose-6-phosphate synthase and one *TPP* gene (Thhalv10025482m) encoding trehalose-6-phosphate phosphatase increased more than two-fold in leaves (Figure 7, Supplementary Table S11). Additionally, raffinose regarded as a compatible solute, antioxidant is involved in stress defense mechanism (ElSayed et al., 2014). Here, two *RFS* genes (Thhalv10005800m and Thhalv10012821m) encoding raffinose synthase and one *GLA* gene (Thhalv10005987m) encoding galactosidase possess significantly elevated expression in leaves, wherein one *RFS* gene (Thhalv10005800m) with ∼12 times enhanced expression (Figure 7, Supplementary Table S11). In short, the transcript abundance of most of *SS*, *TPS*, *TPP*, *RFS*, and *GLA* genes involved in the synthesis of sucrose, trehalose, and raffinose was substantially higher in salt-treated plants than in control, indicating that osmotic adjustment is an important mechanism for *Eutrema* responding to salt shock.

In addition, the expression level of xylose biosynthesis genes *IRX* (Thhalv10012495m) and *XYL4* (Thhalv10003678m), as well as pectin synthesis genes *GAE* (Thhalv10028667m and Thhalv10025193m) and *GAUT* (Thhalv10010596m, Thhalv10006055m, Thhalv10008059m, and Thhalv10004449), increased more than three-fold in leaves after salt stress (Figure 7, Supplementary Table S11). Moreover, the expression of *SORD* (Thhalv10013900m) and *AKR1B* (Thhalv10016976m, Thhalv10016962m, and Thhalv10000256m) genes related to the synthesis of glucose and sorbitol, *lacZ* genes (5) coding galactosidase, and *Pgm* (Thhalv10018328m) and *GALE* (Thhalv10025610m, Thhalv10023559m, and Thhalv10008065m) genes involved in glucose metabolism was also upregulated in leaves. In general, the hydrolysis of starch may be involved in stress-induced response (Krasensky and Jonak, 2012). Our research showed that the level of expression of *GlgA* (Thhalv10006631m and Thhalv10020091m) and *GlgC* (Thhalv10013265m) genes related to the synthesis of amylose and starch was reduced; this was consistent with the general conclusion of a decrease in the starch content under stress (Figure 7, Supplementary Table S11). Collectively, the result revealed that the expression of most genes involved in the synthesis of soluble carbohydrates was upregulated, and the genes related to starch synthesis was downregulated in leaves, which supported that the soluble carbohydrates serving as an osmotic adjustment substance play a critical role in *Eutrema* responding to salt stimulus.

### Contribution of LEA Proteins in Responsive to Salt Stress

The function of LEA proteins has been associated with environmental stress such as drought, salt, and cold, in plants (Tunnacliffe and Wise, 2007). On the basis of domain structures, numbers, and composition of conserved repeat motif, LEA proteins are classified into eight families such as LEA1, LEA2, LEA3, LEA4, LEA5, LEA6, dehydrin, and SMP (seed maturation protein) in the Pfam database (Liang et al., 2016).

**FIGURE 8:**
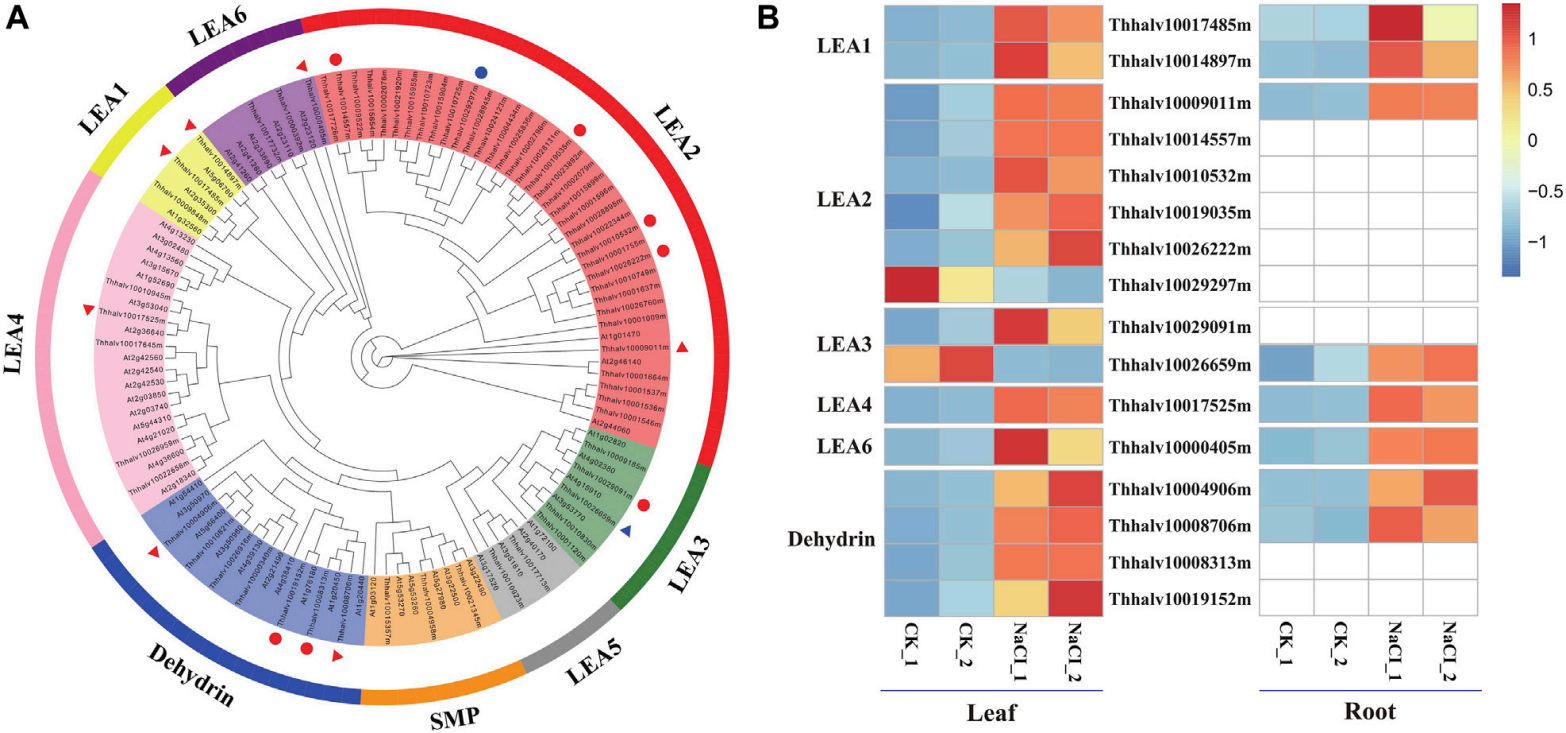
Phylogenetic analysis of LEA proteins and heatmap exhibited the expression of DEGs. **(A)** Phylogenetic analysis of LEA proteins. Red triangles represent the significantly upregulated *LEA* genes both in leaves and roots; blue triangles represent the *LEA* gene markedly downregulated in leaves and upregulated in roots. Red circles indicate significantly upregulated *LEA* genes only in leaves; blue circles indicate markedly downregulated *LEA* genes only in leaves. LEA, late embryogenesis abundant; SMP, seed maturation protein; Dehydrin, dehydrin protein. **(B)** Heatmap analysis of markedly differential LEA genes expression. The transcript levels calculated as RPKM are shown in the color legend.

In order to analyze the contribution of LEA proteins in *Eutrema* upon salt stress, the genome-wide identification of LEA gene families was performed using hmmsearch software combined with the blasting to the homologous genes in *Arabidopsis*. The result showed that a total of 64 LEA proteins identified were classified into eight families, based on the analysis of the conserved domain structures, of which, there were 36 members in LEA2 of the largest families, whereas only two members in LEA5 of the smallest families (Figure 8A). Furthermore, salt stress strongly enhanced the expression of *EsLEA1* (Thhalv10017485m and Thhalv10014897m), *EsLEA4* (Thhalv10017525m), *EsLEA6* (Thhalv10000405m), and *EsDehydrin* (Thhalv10004906m and Thhalv10008706m) genes both in the leaves and roots of *Eutrema*. LEA2 protein members typically accumulated in dehydrating plant seeds and/or in tissues subjected to drought, salinity, and low temperature (Veeranagamallaiah et al., 2011). Dehydrins (DHNs), previously called LEA2, is responsive to ABA; thus, DHNs are also referred to as RAB proteins (Hundertmark and Hincha, 2008). The expression of DHNs could be induced in vegetative tissues by dehydration caused by drought, salinity, and cold (Zhang H.-f. et al., 2020). This study showed that the expression level of genes *EsLEA2* (Thhalv10009011m, Thhalv10014557m, Thhalv10010532m, Thhalv10019035m, and Thhalv10026222m), *EsLEA3* (Thhalv10029091m), and *EsDehydrin* (Thhalv10008313m and Thhalv10019152m) was elevated more than 2 times in leaves (Figure 8B, Supplementary Table S12). Thus, the enhanced expression of LEA2 and Dehydrin protein families, acting as protecting plants from dehydrating damage, was possibly an outstanding response for *Eutrema* encountering salt environment.

## Discussion

Plants are often subjected to high soil salinity during their life cycle (Bressan et al., 2009; Zhao et al., 2020). Understanding the expression changes of co-occurring genes when plants respond to salt is an important aspect for us to explore the mechanisms of retaining plant growth under saline conditions. In this study, RNA-seq analyses were carried out to identify differentially expressed genes in response to salt shock, and we found that most of the genes involved in lignin and soluble sugar biosynthesis as well as scavenging of ROS and oxidized proteins were significantly upregulated; thus, it is proposed that upregulated expression of these genes possibly contributes to the salt tolerance of the halophytic *Eutrema*.

### Transcription Factors Involved in Salt Stress Response

TFs and the corresponding *cis*-acting elements function as molecular switches in the conversion of stress signal perception to stress-responsive gene expression (Zhao et al., 2020). The regulation of gene expression at the transcriptional level is mostly mediated by the sequence-specific binding of transcription factors to the *cis*-acting promoter elements (Kiełbowicz-Matuk, 2012).

In *Arabidopsis*, it has been reported that *AtWRKY11*, *AtWRKY15*, *AtWRKY17*, and *AtWRKY46* are related to the response to salt stress (Vanderauwera et al., 2012; Ding et al., 2015; Ali et al., 2018), and *AtWRKY15* participated in the ROS-induced salt and osmotic stress responses (Vanderauwera et al., 2012)*.* Our RNA-seq result demonstrated that there existed a higher expression of 17 *WRKY* genes in leaves and 3 *WRKY* genes in roots in *Eutrema*, of which the expression of *EsWRKY11* (Thhalv10025646m), *EsWRKY15* (Thhalv10000270m), *EsWRKY17* (Thhalv10000242m), *EsWRKY6* (Thhalv10023390m), and *EsWRKY33* (Thhalv10016542m) was markedly increased more than three-fold in leaves (Figure 4, Supplementary Table S6). Many members of AP2/ERF–ERF superfamily from various plant species reported to be involved in salt and other abiotic stress responses (Baillo et al., 2019). For example, *AtDREB2B* and *AtDREB26* were highly responsive to salt and drought stress (Nakashima et al., 2000; Krishnaswamy et al., 2011), and *AtDEAR4* could play an integrative role in the stress response and senescence via regulating ROS homeostasis (Zhang Z. et al., 2020). In accordance with the previous study, the expression of 15 AP2/ERF–ERF genes including *EsDREB2B*, *EsDEAR4*, and *EsDREB26* was significantly induced in the leaves of *Eutrema* responding to salt in the study (Figure 4, Supplementary Table S6). Additionally, *Arabidopsis AtAF1* gene enhanced the salt tolerance of plants in a previous report (Liu et al., 2016), and our study showed that one NAC family member *EsAF1* (Thhalv10008371m) was upregulated both in roots and leaves upon salt stress, inferring its possible function in salt tolerance.

MYB and bZIP families are associated with different biological activities, including the response to abiotic and biotic stresses (Baillo et al., 2019). In *Arabidopsis*, it was reported that the expression of *AtMYB74* and *AtMYB10*2 was induced upon salt stress (Xu et al., 2015). A similar result that the transcripts of *EsMYB74* (Thhalv10029512m) and *EsMYB102* (Thhalv10027571m) were increased more than 11-fold by salt stress in leaves, whereas no significant expression differences in roots. Meanwhile, the expression of 12 *bZIP* genes was induced and that of 2 was reduced in leaves, while in roots, the expression level of six *bZIP* genes was upregulated and 2 was downregulated (Figure 4, Supplementary Table S6). Furthermore, *EsbZIP1* (Thhalv10005532m) and *EsbZIP9* (Thhalv10004701m) genes were only markedly induced in leaves, which was consistent with the expression of *AtbZIP1 and AtbZIP9* genes previously reported to be enhanced by high-salt stress (Sun et al., 2012; Ortiz-Espín et al., 2017). The members of bHLH and C2H2-type zinc finger families are important TF groups, with the diverse functions in mediating plant stress response (Kiełbowicz-Matuk, 2012). When *Eutrema* was exposed to salt stress, the transcript levels of *EsLRL1-1* (Thhalv10000220m) and *EsLRL1-2* (Thhalv10025656m) being bHLH family were enhanced in leaves, while the expression of six *C2H2* genes including *EsZF2* (Thhalv10021307m) and *EsSTZ/Zat10* (Thhalv10010032m) was also elevated in leaves upon salt stress (Figure 4, Supplementary Table S6).

Abscisic acid (ABA) could mediate numerous physiological processes and regulate the adaptive stress responses including the exclusion of excessive salts (Keskin et al., 2010). Some members of AP2/ERF–ERF, MYB, WRKY, bZIP, and NAC families have been reported to be regulated by ABA signaling in *Arabidopsis* and are associated with abiotic stress defense responses in plants (Zheng et al., 2019). In this study, the expression levels of 20 TF genes were markedly increased upon salt stress, such as *EsDREB2B*, *EsDEAR4*, *EsDREB2*6, and *EsERF8* of DREB superfamily; *EsMYB74*, *EsMYB102*, *EsMYB94*, *EsDIV2*, and *EsMYB49* of MYB family; *EsABI5*, *EsGBF3*, *EsABF3*, and *EsABF*2 of bZIP family; *EsWRKY6*, *EsWRKY33*, *EsWRKY11*, and *EsWRKY17* of WRKY family; and *EsRD26*, *EsAF1*, *EsNAC47*, of NAC family, in leaves and/or roots (Figure 4B, Supplementary Table S13). The promoter element analysis indicated that the promoters of these TF genes all contain ABA-responsive *cis*-elements using the PlantCare website (http://bioinformatics.psb.ugent.be/webtools/plantcare/html/) (Supplementary Table S13). Furthermore, according to the homologous genes previously reported in *Arabidopsis* (Zheng et al., 2019), it is inferred that the above 20 TF genes possibly participate in response to ABA.

In summary, salt stress led to a significant expression change of many TF members in WRKY, AP2/ERF–ERF, NAC, MYB, bZIP, and HSF families in leaves and/or roots, and the expression patterns of most numbers of TF families were different between the leaves and roots. Moreover, some transcription factor genes are likely involved in the ABA signaling pathway.

### Enhancing Antioxidant Activity and Osmotic Protection Capacity Suggested Playing Key Roles in Response to Salt Stress in *Eutrema*

In general, plant cell walls suffer lignification during stress (Dong and Lin, 2021). Lignin, as a polyphenolic biopolymer, facilitates hydrophilic transport and contributes to cell rigidity (Boerjan et al., 2003). Our transcriptome analysis illustrated that the expression of 41 DEGs possibly involved in lignin biosynthesis was significantly influenced by salt shock, including genes of *EsMYB15*, *PAL*, *C4H*, *C3′H*, *COMT*, *CCoAOMT*, *HCT*, *4CL*, *CCR*, *CAD*, and *POX/POD* (Figure 5A and Figure 5B, Supplementary Table S7). The lignification of the cell wall facilitates water transport of plants; thus, we further detected the lignin content, and the result demonstrated that 300 mM NaCl stress significantly enhanced lignin accumulation in *Eutrema* leaves and roots from 10-day salt-treated plants. Therefore, it is suggested that lignin biosynthesis could provide contribution for the halophytic *Eutrema* to respond to salt stress.

ROS production can be enhanced by many abiotic stresses, such as salt, drought, and high light (Van Breusegem and Dat, 2006). In leaves of *Arabidopsis* and *Eutrema*, irrigated with 0.15 and 0.3 M NaCl, respectively, the result documented an enhanced content of H_2_O_2_ in *Eutrema* compared to that in Arabidopsis after 7 days of treatment (Pilarska et al., 2016). However, a very low level of malondialdehyde (MDA) was detected in *Eutrema* leaves, indicating a low extent of oxidative damage to membrane lipids (Pilarska et al., 2016). Autophagy is associated with a wide range of stress responses through degrading oxidized proteins under stress conditions (Xiong et al., 2007a; Xiong et al., 2007b). In the study, there were six *ATG* and four *Vac8* genes identified, and the markedly induced gene expression of *ATG3* (1), *ATG4* (1), *ATG7* (1), *ATG8* (3), and *Vac8* (4) revealed that autophagy could play an important role in eliminating oxidized proteins caused by salt stress in *Eutrema* (Figures 6A–C, Supplementary Table S5 and Supplementary Table S9). In the peroxisome pathway, 15 DEGs, involved in matrix protein import, ROS metabolism, antioxidant system, and fatty acid oxidation, exerted significant changes upon salt stress (Figure 6D and Figure 6E, Supplementary Table S10). The result implied an increased expression of genes encoding transport proteins in the peroxisome membrane, antioxidant enzymes in the matrix, and genes involved in the α/β-oxidation of fatty acid oxidation, which is possibly a way for *Eutrema* to cope with salt stress via scavenging ROS generated by salt stress.

In addition, approximately 45 DEGs mainly participated in sugar metabolism, including galactose metabolism, starch and sucrose metabolism, and fructose and mannose metabolism, of which the expression of 35 DEGs were markedly upregulated in leaves (Figure 7, Supplementary Table S11). Salt-treated plants usually need a higher capacity to adjust osmotically to cope with salt stress (Krasensky and Jonak, 2012). Thereby, sugar metabolism pathways associated with osmotic adjustment are of great significance for the osmotic protection of *Eutrema*. In contrast to other proteins involved in desiccation tolerance caused by drought and salt, LEA proteins have no apparent enzymatic activity and might act as the “molecular shield function” to protect enzymes from induced aggregation under stress conditions (Goyal et al., 2005). LEA proteins also aid in the formation of the glassy state, in which nonreducing sugars accumulate in the cytoplasm of plants during periods of desiccation (Shimizu et al., 2010). For example, the expression of HVA1, an LEA3 protein from barley (*Hordeum vulgare* L.), conferred tolerance to salt stress in transgenic rice plants (Xu et al., 1996). Although LEA proteins are mainly observed in seeds, they have also been detected in seedlings, buds, and roots of plants (Hundertmark and Hincha, 2008). In our research, there was markedly induced expression of *EsLEA1*, *EsLEA4*, *EsLEA6*, and *EsDehydrin* genes in leaves and/or roots (Figure 8, Supplementary Table S12), implying the accumulation of LEA proteins is an important strategy for *Eutrema* to deal with salt stress.

**FIGURE 9:**
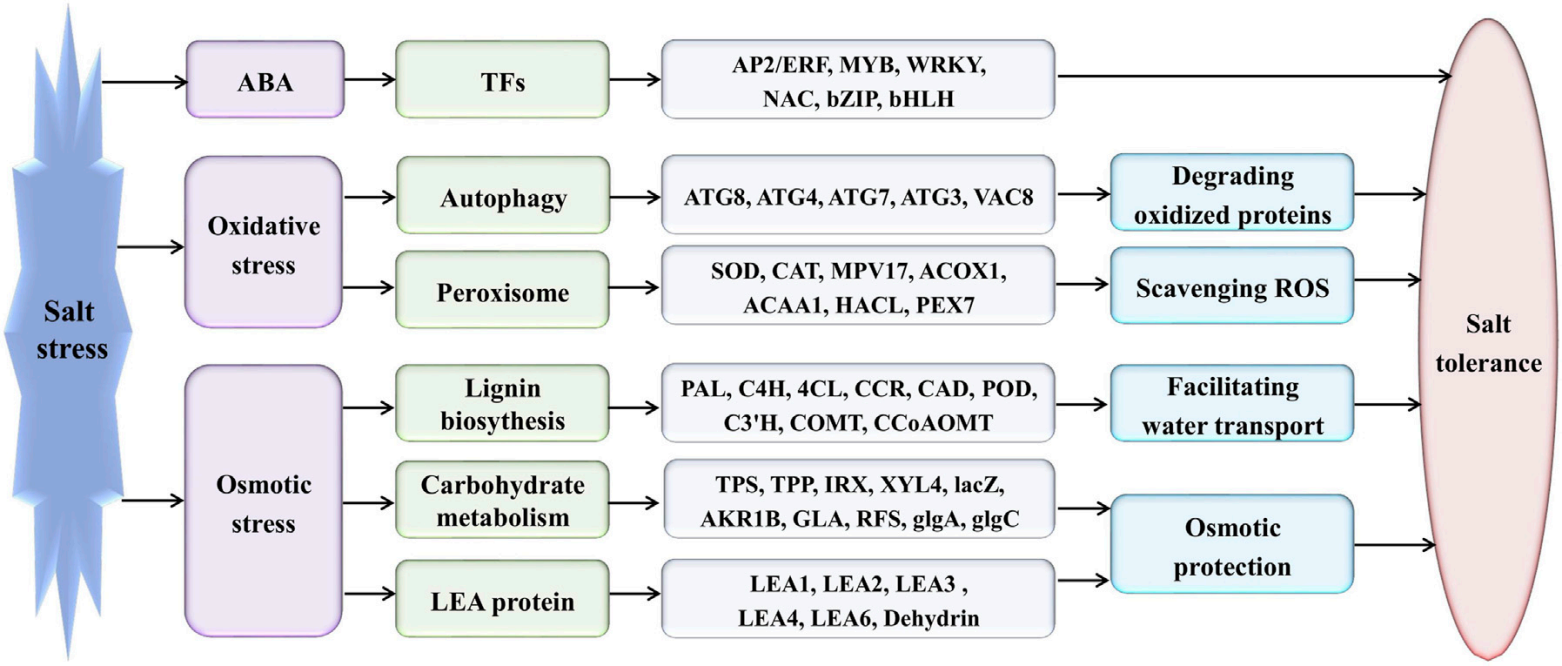
Proposed model for the mechanism of *Eutrema* responding to salt stress. AP2/ERF, APETALA 2/ethylene-responsive element binding factor; MYB, v-myb avian myeloblastosis viral oncogene homolog; WRKY, WRKY transcription factors; NAC, NAM, ATAF and CUC family; bZIP, basic leucine zipper; and bHLH, basic helix-loop-helix. ATG, autophagy-related protein; VAC, vacuolar membrane protein; SOD, superoxidase dismutase; CAT, catalase; MPV17, Mpv17 protein; ACOX, acyl-coA oxidase; ACAA, acetyl-CoA acyltransferase; HACL/HPCL, 2-hydroxyacyl-CoA lyase; PEX, peroxin; PAL, phenylalanine ammonia-lyase; C4H, cinnamic acid 4-hydroxylase; 4CL, 4-coumaric acid: CoA ligase; CCR, cinnamoyl CoA reductase; CAD, cinnamyl alcohol dehydrogenase; POD/POX, peroxidase; C3′H, *p*-coumaroyl shikimate 3′-hydroxylase; COMT, caffeate 3-O-methyltransferase; CCoAOMT, caffeoyl-CoA O-methyltransferase; TPS, trehalose-6-phosphate synthase; TPP, trehalose-6-phosphate phosphatase; IRX, 1,4-beta-D-xylan synthase; XYL4, xylan 1,4-beta-xylosidase; lacZ, beta-galactosidase; AKR1B, aldehyde reductase; GLA, alpha-galactosidase; RFS, raffinose synthase; glgA, glycogen synthase; glgC, glucose-1-phosphate adenylyltransferase; LEA, Late embryogenesis abundant; Dehydrin, dehydrin protein.

*Eutrema* tolerates extreme salinity, cold, drought, ozone (Inan et al., 2004; Li et al., 2006; Amtmann, 2009; Hou and Bartels, 2015). So far, the studies on the transcript profile in *Eutrema* found that several stress-associated genes have a constitutively higher expression in the absence of stress (Inan et al., 2004; Taji et al., 2004; Gong et al., 2005). For instance, Fe-SOD, P5CS, PDF1.2, AtNCED, P-protein, b-glucosidase, and SOS1 were constitutively expressed in salt cress at high levels even in the absence of stress (Taji et al., 2004). In our research, the similar “stress preparedness” was found, such as proline biosynthesis key enzyme encoding genes *P5CS* (Thhalv10016322m, Thhalv10010150m), ABA biosynthesis key enzyme encoding gene *NCED3* (Thhalv10020337m), glycine decarboxylase P-protein encoding genes (Thhalv10024257m, Thhalv10001891m), a plasma membrane Na^+^/H^+^ antiporter *SOS1* (Thhalv10003547m), a vacuolar Na^+^/H^+^ antiporter *NHX1* (Thhalv10003949m), a controlled lateral root formation *TCTP1* (Thhalv10021661m), aquaporin *PIP1A* (Thhalv10001573m), and *PIP2A* (Thhalv10010598m), all possessing constitutively higher expression under normal growth conditions without salt stress (Supplementary Table S14). Anyway, the significantly differentially expressed genes affected by salinity were enriched in the pathways of lignin biosynthesis, autophagy, peroxisome, and sugar metabolism, indicating the related metabolism processes could play critical roles in response of *Eutrema* to salt stress (Figure 9).

### Effect of Salt Shock on Genes Associated With Photosynthesis and Ribosome

**FIGURE 10:**
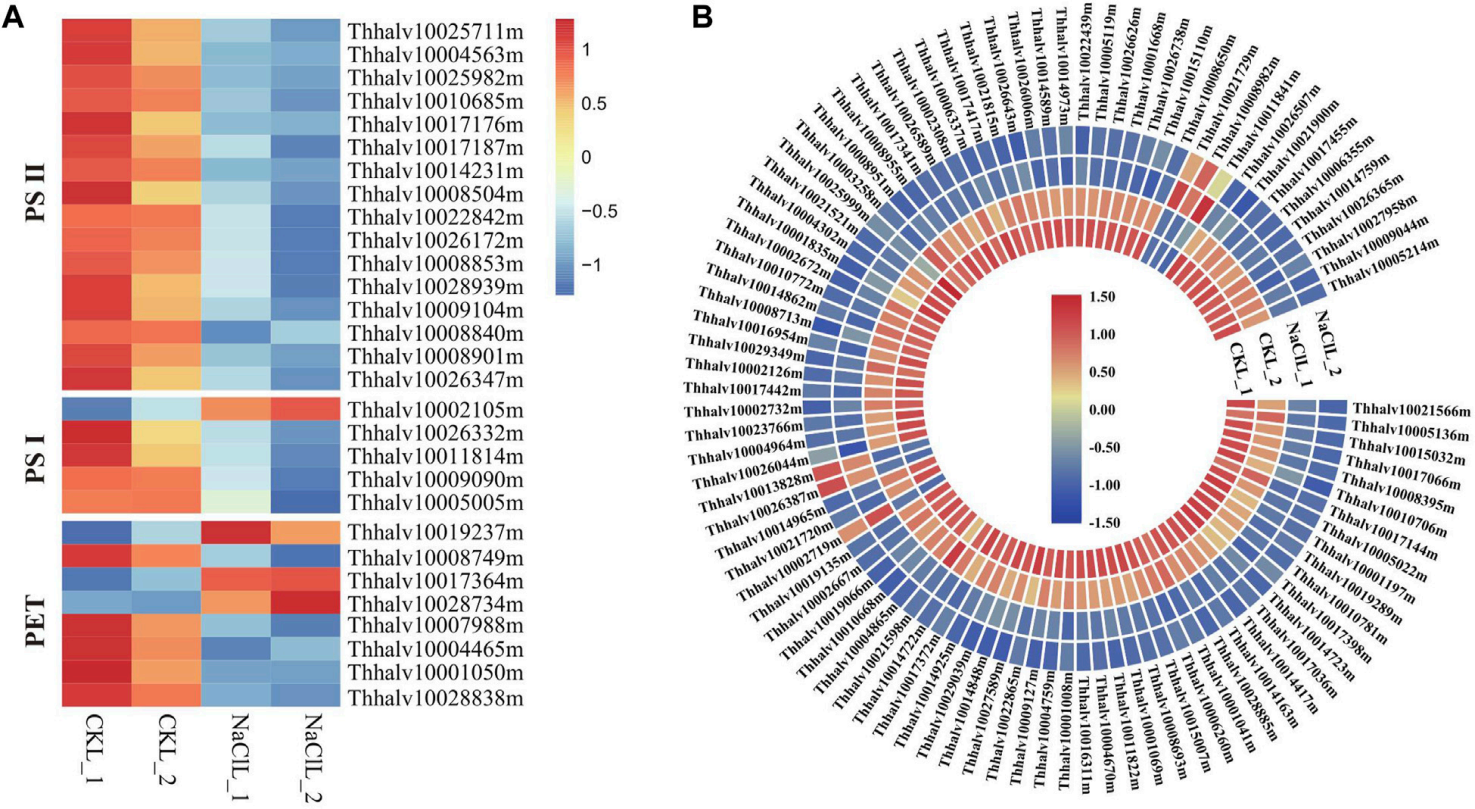
DEGs enriched in pathways of photosynthesis and ribosome in leaves. **(A)** Heatmap analysis of DEGs enriched in the photosynthesis pathway. PET indicates the photosynthetic electron transport. The transcript levels calculated as RPKM are shown in the color legend. **(B)** Heatmap analysis of DEGs enriched in the ribosome pathway. The transcript levels calculated as RPKM are shown as in the color legend.

Photosynthesis is the primary processes affected by water or salt stress (Chaves et al., 2009); thus, considerable attention has been previously paid to the effects of environmental stress on these photosystems (Allakhverdiev and Murata, 2008). The efficiency of photosynthesis is closely related to the activities of photosystem II (PSII) and photosystem I (PSI), converting light energy to chemical energy via electron transport (Nishiyama et al., 2005). PSII and, sometimes, PSI is particularly sensitive to environmental stress (Allakhverdiev and Murata, 2008); thus, the effects of salt are critical in salt-sensitive species, whereas salt-tolerant species can protect the formation of photosynthetic assemblies by increasing the expression of particular genes (Yang et al., 2020). Analyzing *Eutrema* transcriptome data revealed that a total of 29 DEGs in leaves are enriched in the photosynthesis pathway (ko00195). The proteins encoded by these DEGs are mainly involved in photosystem I, photosystem II, and photosynthetic electron transport (PET). Genes involved in photosystem II in leaves including *PsbO* (2), *PsbP* (6), *PsbQ* (4), *PsbW* (1), *Psb27* (2), and *Psb28* (1) were all downregulated 0.33–0.62 times, and the expression of *PsaE* (Thhalv10026332m), *PsaH* (Thhalv10011814m), *PsaK* (Thhalv10009090m), and *PsaN* (Thhalv10005005m) in photosystem I was declined 0.5–0.57 times in leaves (Figure 10A, Supplementary Table S15A). There were smaller changes in gene expression related to photosynthesis in the leaves, whereas no significant difference in the genes related to photosynthesis in roots of the salt treatment group compared with the control (Supplementary Table S15A); thus, it is suggested that the salt stress of 300 mM NaCl for 24 h could be a milder stress for the halophytic *Eutrema*. Additionally, we found that the expression of genes related to photosynthesis was obviously lower in roots than in leaves (Supplementary Table S15A), which is in accordance with the conclusion that photosynthesis usually takes place only in the aboveground tissues of the plants.

Ribosomal proteins are essential for proper growth and development of any organism (Mukhopadhyay et al., 2011). As a general trend, salt stresses lead to the downregulation of ribosomal protein–encoding genes, which was consistent with our analysis in leaves of *Eutrema*. Here, 97 differentially expressed genes were significantly enriched in the ribosome pathway (ko03010), of which the expression of 90 DEGs such as *EsL10e* (Thhalv10025999m and Thhalv10003258m), *EsL15e* (Thhalv10027381m), *EsL24e* (Thhalv10017341m), and *EsL30* (Thhalv10026738m) was downregulated upon salt stress (Figure 10B, Supplementary Table S15B), indicating that the imposed influence of salt stress on genes associated with the ribosome protein. Overall, although *Eutrema* is a halophyte possessing a higher salt tolerance, salt stress still imposed some effects on photosynthesis and ribosome.

## Conclusion

In summary, salt shock led to a large number of significantly differentially expressed genes enriched in autophagy (*Vac8*, *Atg8*, and *Atg4*), peroxisome (*PEX7*, *CAT*, and *SOD2*), lignin biosynthesis (*CCoAOMT*, *C4H*, *CCR*, *CAD*, *POD,* and *C3′H*), and sugar metabolism (*SS*, *TPS*, *TPP*, and *RFS*) pathways, and these DEGs were mainly involved in the scavenging of ROS and oxidized protein, as well as the biosynthesis of lignin and soluble sugar, indicating that avoiding oxidative damage and osmotic adjustment are the key mechanisms for *Eutrema* in response to salt stress. This study helps improve our understanding about salt cress responding to salt shock and its mechanism of salt tolerance. Therefore, this research lays an important foundation to provide the excellent gene resource for genetic breeding to boost the salt tolerance of crops.

## Data Availability

The datasets presented in this study can be found in https://www.ncbi.nlm.nih.gov/bioproject/, and the accession number of the NaCl treatment and control groups were PRJNA737318 738 (SRR14804235‐SRR14804238) and PRJNA438390 (SRX3793601–SRX3793604), respectively.
